# Electrode Afterload: A Valuable Technique in a Case of Short Electrode Insertion

**DOI:** 10.1155/2020/3910138

**Published:** 2020-02-13

**Authors:** C. Riemann, S. Scholz, H. Sudhoff, I. Todt

**Affiliations:** ^1^Department of Otolaryngology, Head and Neck Surgery, Bielefeld University, Klinikum Bielefeld, Campus Mitte, Bielefeld, Germany; ^2^Department of Auditory Rehabilitation, Oberlinhaus Rehazentrum, Potsdam, Germany

## Abstract

*Introduction*. The location of the electrode inside the cochlea is important for speech performance. However, many variables, including array length, insertion depth, and individual anatomy, may affect the intracochlear position of the electrode. Insertion deeper than 20 mm and revision surgery are critical situations in which residual hearing and electrode integrity may be at risk. This case report challenges this hypothesis and raises the following question: is it possible to achieve a better speech understanding with an electrode afterload without compromising residual hearing? *Case Report*. A 73-year-old female patient showed up for evaluation of hearing loss. The patient was operated four times in an external hospital due to cholesteatoma formation in the right ear. Related to a poor aided speech understanding, a CI-surgery was performed. 5 months after the surgery, the subject returned with poor speech understanding. A revision surgery was performed, where the first white marker of the electrode was seen in the round window (20 mm). The electrode was inserted 4 mm deeper into the cochlea. After six and twelve months, the results of the Freiburger monosyllabic speech test improved till 25% and 45%, respectively. *Discussion*. Hearing preservation is possible with a revisional deeper insertion from 20 mm to 24 mm. In this case, a partial obliteration of an open cavity made the electrode surgically easily accessible. This allowed the deeper insertion during the revision surgery. In a regular surgical field with a posterior tympanotomy, the revision surgery is more challenging and brings the electrode into the risk of an iatrogenic destruction. *Conclusion*. This case of an electrode afterload after having inserted the electrode initially to mm, demonstrates that hearing can be preserved and speech perception can improve after performing this maneuver.

## 1. Introduction

Electrode technology and its interaction with the human cochlea continue being one of the most studied and researched topics in modern otology. Recently, shorter straight electrodes for combined electric and acoustic stimulation (hybrid) were introduced to the market [1]. The benefits of this type of stimulation have been established, for which electrode design is critical for preservation of residual hearing. A number of electrodes suited to hybrid hearing have been developed over the past 15 years. These electrodes have been straight with limited length to avoid intracochlear trauma associated with very deep insertion [2, 3]. Electroacoustic stimulation is a promising treatment for individuals with too much residual hearing to receive a cochlear implant for pure electrical stimulation but not enough hearing to benefit from a pure hearing aid. In this treatment, an electrode array provides electric stimulation for high-frequency sounds, whereas the listener uses their residual hearing for low-frequency aided hearing [4].

In order to facilitate the hybrid stimulation, the location of the electrode in relation to the cochlea is important for speech performance. Current electrode designs attempt to replicate natural cochlear tonotopic organization by delivering low-frequency information to apical electrodes and high-frequency information to basal electrodes. However, many variables may affect the absolute placement of the electrode including array length, electrode spacing, surgical technique, insertion depth, and individual cochlear anatomy [5]. The optimum electrode array length and the target insertion depth to achieve have been a subject of much debate in terms of benefits in speech recognition and how well hearing may be preserved. Previously, it was suggested to use a relatively short 10 mm hybrid electrode array to maximize the chance of preserving residual hearing. Nowadays, this principle has been extended to either 16 mm or 20 mm lateral-wall electrodes [6]. Insertion deeper than 20 mm is a critical period during which the hearing may be at risk. Despite all efforts to achieve a complete hearing preservation (defined as a hearing within 10 dB of the preoperative level), it is accomplished in only one third of patients [7]. Others follow a concept of an initial increased cochlear coverage (28 mm) with good results in speech understanding [8].

Another situation, in which hearing may be at risk, is revision surgery (electrode reimplantation). The most common indication for revision surgery is device failure [9]. This procedure may injure the anatomical structures of the cochlea, resulting in loss of residual hearing [10]. Although revision surgery is an undesirable consequence of cochlear implantation, some recent studies have shown good results in terms of speech perception and preservation of residual hearing [11]. Another objective of revision surgery is optimizing insertion depth. Insertion depths may vary depending on the amount of residual hearing to be preserved. Deep insertions are usually associated with cochlear injuries and loss of residual hearing [12]. This case report challenges this hypothesis and proposes the following question: is it possible to achieve a better speech perception with an electrode afterload by inserting it deeper than 20 mm without compromising residual hearing or the integrity of the array?

## 2. Case Report

A 73-year-old female patient came to our clinic for evaluation of hearing loss. The patient had a previous history of chronic otitis media mesotympanalis with cholesteatoma formation in the right ear. Due to this reason, the subject was operated four times in an external hospital. In the first surgery, the cholesteatoma was removed and a mastoid open cavity was created. The following surgeries were aimed at a second look and hearing improvement. Unfortunately, an improvement of the hearing could not be obtained. The patient has been wearing a hearing aid (Phonak Naida V) in the right ear since then with decreasing satisfaction and did not want any revision surgery but a definitive solution for the hearing loss. In 2010, the patient received a cochlear implant (Nucleus CI-512) in the left ear due to a progressive hearing loss with functional deafness. The patient was very satisfied with the results in the left ear; therefore, the subject showed up at our department with the question whether one device could improve her symptoms in the right ear.

**Figure 1:**
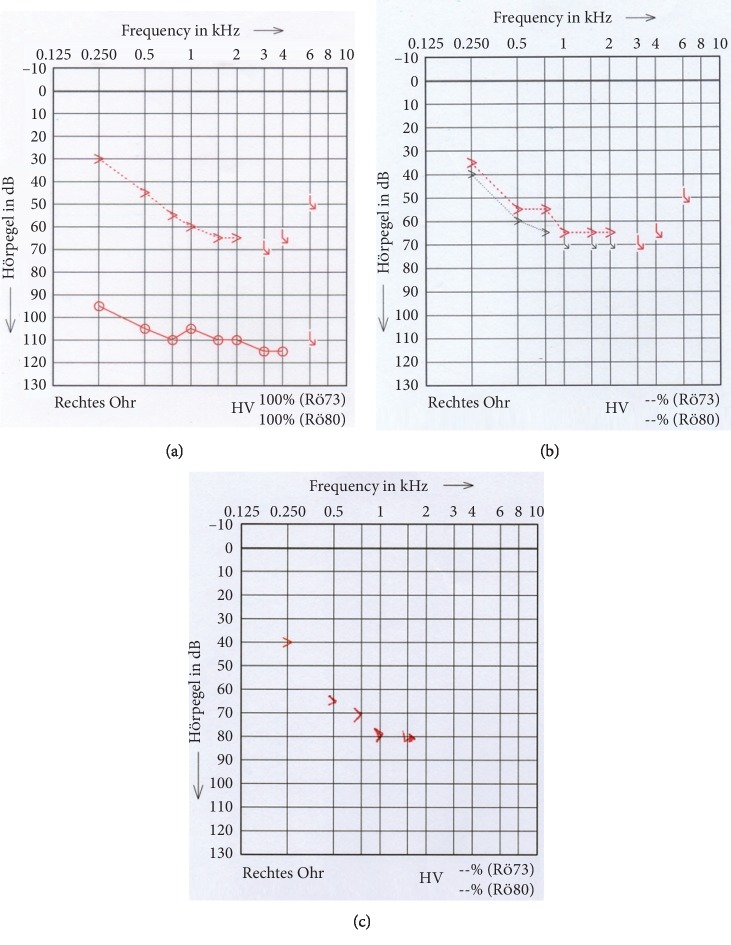
(a) First PTA. (b) PTA after first surgery. (c) PTA after revision surgery.

The neurotologic physical examination was normal. An audiogram was performed, which demonstrated a combined hearing loss with a diagonal decay from 30 dB and an air-bone gap from approximately 65 dB in the right ear (Figure 1(a)). The Freiburger monosyllabic speech test in a quiet environment at 65 dB was performed in the right ear with the hearing aid and in the left ear with the cochlear implant. The subject scored 10% in the ear to be implanted and 75% in the contralateral ear. The computerized tomography of the right temporal bone and the magnetic resonance imaging of the right inner ear were normal. On the basis of the audiogram, speech perception results, and demographic factors, the subject fitted the criteria for a cochlear implant in the right ear. According to the audiogram, the subject had still an aidable low-frequency hearing; therefore, it was decided that an electroacoustic stimulation would benefit her the most. The Nucleus slim straight electrode (CI-522) was chosen.

**Figure 2:**
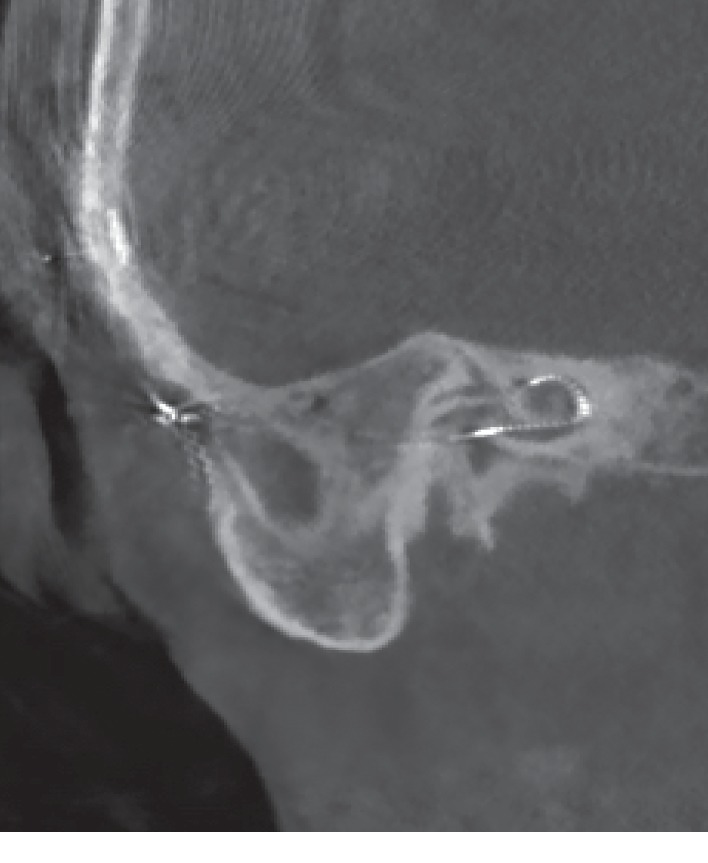
CT after first surgery.

The surgery was performed in a standard fashion: a retroauricular skin incision was made on the right side, the planum mastoideum was presented, and the epithelium to the tympanic cavity was lifted. In the tympanic cavity, a PORP with granulations was seen. The round window was located, and the implant bed was then created. The round window was carefully opened and the slim straight electrode was inserted until the first marker was seen in the round window. The electrode was fixed in the mastoid cavity using fibrin glue and bone-meal. Prednisolon (1gr) and Ceftriaxone (2gr) were systemically administered intraoperatively. The final measurements of the impedances and NRT-potentials yielded favorable results. The patient tolerated the procedure well and without complications. A computerized tomography of the temporal bone was made to verify the position of the electrode after surgery. The CT showed an insertion depth of about 290° (Figure 2). Two days after surgery, the patient was discharged.

The subject returned one month after surgery for her device activation. The four-week unaided audiogram revealed good preservation of residual hearing (Figure 1(b)). The electrical stimulation was initiated. Five months after surgery, the patient returned to us with poor speech understanding and sound quality in the implanted ear. According to the patient, the hearing had remained the same as before the surgery. The Freiburger monosyllabic speech test in a quiet environment in the right ear with the cochlear implant was once again performed. This time, the subject scored 0%. There were no signs of device failure. There was no history of infection or head trauma in the past months. To discard a displaced implant, a CT-scan was performed and indicated that the cochlear implant was in a normal position in the basal turn of the cochlea. Due to the unfavorable results, a revision surgery was proposed. The patient agreed.

**Figure 3:**
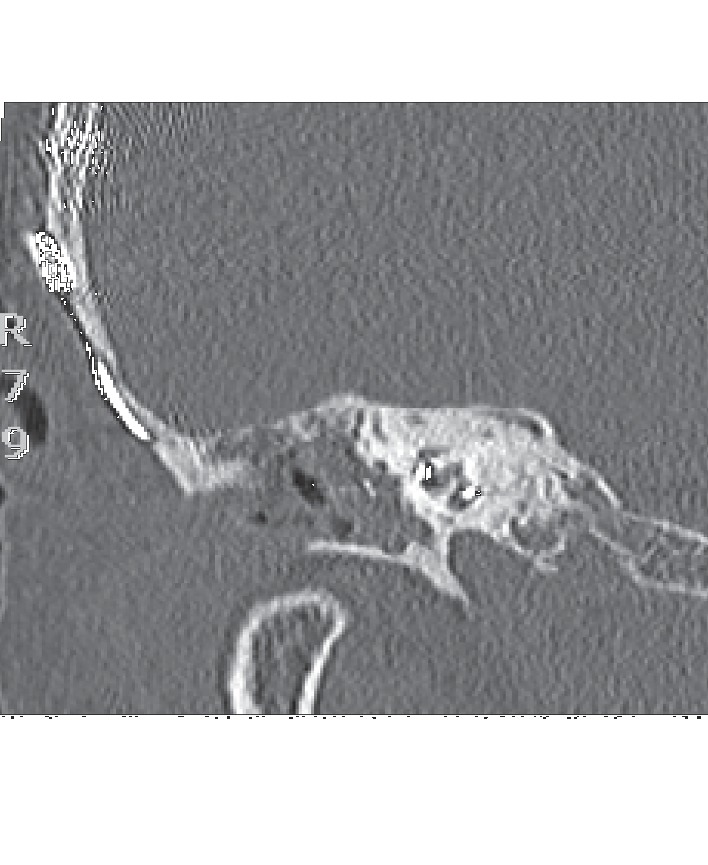
CT after revision surgery.

For the revision surgery, an endaural approach was performed. A tympanomeatal flap was made, and the cartilage covering the open cavity was lifted. The electrode was visualized, and the scar-tissue around it was removed. The first marker of the slim straight electrode was once again seen in the round window. The electrode was inserted approximately 4 mm deeper into the cochlea until just before the second marker was seen in the round window. The electrode was fixed again, and the NRT-potentials showed normal results. The surgery and the hospital stay were without complications. A CT-scan of the temporal bone was performed, and an insertion depth was reported from approximately 380° (Figure 3).

Three months after the revision surgery, the subject described a subjective hearing improvement. Once again, the audiogram showed good preservation of residual hearing (Figure 1(c)). The Freiburger monosyllabic speech test in a quiet environment was repeated and showed an improvement up to 25%. After six and twelve months, the results were 25% and 45%, respectively. This case report is unique in the literature because it demonstrated that, after introducing the electrode more deeply into the cochlea, the residual hearing was preserved and the speech perception improved significantly, contrary to what has been published.

## 3. Discussion

It was once thought that only shorter electrodes with short insertion depth had the ability to preserve residual hearing. On the other hand, the short insertion depth has been correlated with poor speech recognition. Over time, longer electrodes with greater insertion depth have been developed and continued to show favorable results in terms of hearing preservation and speech understanding [4, 13]. These two variables should be carefully balanced when choosing an electrode array. Fitzgerald et al. reported better speech understanding in a case series of two subjects, after replacing a 10 mm electrode with a full-length array. Unfortunately, both of them had a loss of residual hearing after the first implantation [4]. Dunn et al. also described significant improvements after replacing a 10 mm electrode with a 16 mm array, while preserving residual hearing [13]. These results suggest the possibility of using longer arrays in hybrid devices to begin with, thus avoiding the need for afterloading.

Hearing preservation is possible with a revisional deeper insertion from 20 mm to 24 mm [14]. The CI-522 has a total length of 25 mm with 22 half-band electrodes spread over 20 mm. In a case series of 35 subjects implanted with the CI-522, residual hearing was well conserved. This suggests that there was little trauma to the cochlea, even with deeper insertion depths [6]. In another study with the CI-522, continuous real-time response telemetry was applied while inserting the array. This study suggested that the array can be inserted to 20 mm (the first white marker) without functional evidence of trauma and that for insertions beyond 20 mm, the array should be advanced with meticulous care. Acoustic hearing was most at risk during the last few millimeters of the insertion [7]. In our study, hearing was well preserved, even with an insertion greater than 20 mm.

Hearing loss is progressive, and the patients implanted with a hybrid device then have to rely on shallower insertion depths for electric stimulation only. Following this concept, an electrode afterload seems attractive and logical when hearing loss progresses and greater insertion depths are required for better outcomes. Also, in the case of postoperative hearing loss, the electrode can be further inserted, so the patients can benefit from greater cochlear coverage using electric stimulation only [10].

During revision surgery, mechanical trauma to the device must be avoided at all time. Normally the electrode array may have integrated soft tissue, and inadvertent drill contact with adherent soft tissue may damage the seal from the device. In a regular surgical field with a posterior tympanotomy, an afterload of the electrode might be more challenging and brings the electrode into the risk of an iatrogenic destruction. In this particular case, a partial obliteration of an open cavity made the electrode surgically easily accessible. This allowed an easy deeper insertion during the revision surgery, without compromising the integrity of the device.

Electrode migration or partial insertion during the first surgery could be the causes of no improvement in speech perception [10]. After the revision surgery, it could be confirmed that the electrode did not migrate outside the cochlea and that it was fully inserted (first white marker was seen in the round window). One suggested hypothesis of why speech perception got better after deeper insertion is cochlear tonotopicity with neural survival. In this patient, neural survival in the base of the cochlea may have been poorer than in the most apical parts, causing an improvement in speech perception when the electrode was inserted deeper [4]. The new electrode position produced a greater degree of cochlear coverage in the apical regions and, thus, a better tonotopic place representation for stimulation [15]. Recent studies of Flex 28 electrodes underline the advantage of an increased coverage of the cochlea [8]. Some groups showed no correlation between deeper insertion depths and hearing preservation [16–18]. Selection of the optimal length of the electrode and insertion depth is complex and should be personalized [6].

## 4. Conclusion

This unique case report of an electrode afterload after having inserted the electrode completely demonstrates that the cochlea might be more robust than once thought, since hearing was preserved and speech perception improved after performing this maneuver.
